# HY Immune Tolerance Is Common in Women without Male Offspring

**DOI:** 10.1371/journal.pone.0091274

**Published:** 2014-03-19

**Authors:** Miranda P. Dierselhuis, Ewa Jankowska-Gan, Els Blokland, Jos Pool, William J. Burlingham, Astrid G. S. van Halteren, Els Goulmy

**Affiliations:** 1 Dept. of Immunohematology and Blood Transfusion, Leiden University Medical Center, Leiden, The Netherlands; 2 Dept. of Surgery, University of Wisconsin, Madison, Wisconsin, United States of America; 3 Immunology Laboratory/Dept. of Pediatrics (WAKZ), Leiden University Medical Center, Leiden, The Netherlands; Fujita Health University, School of Medicine, Japan

## Abstract

**Background:**

Sex difference is an established risk factor for hematopoietic stem cell transplantation (HSCT)-related complications like graft versus host disease (GVHD). CD8^pos^ cytotoxic T cells specific for Y chromosome-encoded minor Histocompatibility antigens (HY) play an important role therein. Prior to HSC donation, female donors may encounter HY antigens through fetomaternal or transmaternal cell flow, potentially leading to the induction of HY-specific cytotoxic or regulatory immune responses. Whether HY priming occurs independent of parity, and whether HY priming is dependent on the presence of male microchimerism, is as yet unknown.

**Methods:**

We investigated the presence of HY-specific regulatory T cells (Treg) and male microchimerism in 45 healthy women with a fully documented pregnancy and family history. HY peptide-induced linked suppression, a commonly reported functional feature of CD4^pos^ and CD8^pos^ Treg, was measured by *trans vivo* Delayed Type Hypersensitivity testing. As source of HY antigens, male microchimerism was analyzed by real-time PCR and defined by the presence of male DNA in at least one purified leukocyte cell type.

**Results:**

HLA class I or class II restricted HY-specific Treg were detected in 26/42 (62%) women eligible for analysis. The prevalence of HY-specific Treg was significantly higher in women who had never given birth to sons than in women with male offspring (p = 0.004). Male microchimerism could be detected in 24 out of 45 (53%) women but did not correlate with the presence of HY specific Treg.

**Conclusions:**

HY-specific Treg in women with male offspring have been described previously. Here we show for the first time that, in fact, HY specific Treg are more common in nulliparous women and in parous women with female offspring. Their presence is independent of the presence of male microchimerism. Whether HY-specific Treg presence in female stem cell grafts might decrease the GVHD incidence in male HSCT recipients needs to be investigated.

## Introduction

Sex mismatching is often associated with life-threatening transplantation-related complications [1]. Female donor-derived T cells present in hematopoietic stem cell grafts are involved in the immunopathology of Graft *versus* Host Disease (GVHD) in male hematopoietic stem cell transplantation (HSCT) recipients. CD8^pos^ HLA/HY tetramer-binding T cells have been detected prior to HSCT in peripheral blood of female donors and have been visualized after HSCT in peripheral blood of male recipients [2]. Furthermore, CD8^pos^ HY-specific T cells could be demonstrated in skin biopsies of male recipients who developed GVHD early after infusion of a female donor stem cell graft [3].

Cytotoxic T lymphocytes (CTL) specific for autosomal or Y chromosome-encoded minor H antigens are also induced in the physiological setting of pregnancy. During fetal development, HY-specific T cell responses become detectable from the first trimester [4] and continue to persist for many years post-partum [4]–[7]. It is assumed that trans-placental passage of male leukocytes (designated as fetomaternal cell flow) is the most likely scenario for the priming of these HY-specific immune cells.

During pregnancy, not only maternal- and child-derived cells are mutually exchanged but also trans-maternal cell flow does occur. Herein, cells from a former pregnancy or from a twin sibling who died *in utero* (‘vanished’ twin) can be transferred during the next pregnancy [8]–[10]. Indeed, minor H antigen-specific immune responses to trans-maternally transmitted cells have been recently demonstrated. Namely, HY-specific CTL were detected in cord blood samples of newborn girls with older brothers [9]. Interestingly, these HY-specific CTL could also be demonstrated in umbilical cords of newborn girls without older brothers, indicating that other sources of male cells in the maternal circulation can induce minor H antigen-specific responses [9].

Before, we demonstrated that not only CD8^pos^ CTL but also CD8^pos^ T cells with an immune suppressive function specific for autosomal or for Y chromosome-encoded minor H antigens, were observed in a small group of multiparous women [7]. The latter regulatory T cells (Treg) displayed bystander immune suppression in a CTLA-4-dependent fashion when tested in the *trans vivo* Delayed Type Hypersensitivity (tvDTH) assay. In the latter study, the role of microchimeric cells, possibly instrumental for the induction of minor H antigen-specific T cells, was not investigated.

To get insight into the spectrum of minor H antigen-specific immune responses putatively present in HSCT female donors, we studied, as proof of principle, Treg responses against HY minor H antigens. Forty-five healthy females were analyzed for the presence of Treg against 8 different minor HY antigens; their presence was analyzed in relation to male microchimerism (MC^Y-gene^).

## Materials and Methods

### Ethics Statement

Peripheral blood samples and detailed information on obstetric and family history were obtained from 45 healthy, non-transfused, women after receiving written informed consent according to the declaration of Helsinki. Approval for this study (P06.008) was obtained from the Institutional Review Board of the Leiden University Medical Center.

### Study participants

The included women expressed at least one of the HY peptide-presenting HLA class I alleles (HLA-A1, HLA-A2, HLA-B7, HLA-B8 or HLA-B60) and/or HLA class II alleles (HLA-DR15, HLA-DQ5 or HLA-DRB3*0301) (Table 1). All analyses were performed on aliquoted samples prepared from the buffy coat of a 450 ml whole blood donation.

**Table 1:** Overview of HLA class I and II restriction molecules, obstetric and family history, male microchimeric cell types and HLA class I and II HY-specific T regulator cells.

ID	HY peptide presenting HLA class I allele(s)	HY peptide presenting HLA class II allele(s)	G*	Sons	elder brother	MC^Y-gene^ (cell subset)	% HY^class-I^ induced inhibition@	% HY^class-II^ induced inhibition	% HX^class^ ^I+II^ induced inhibition
♀1	A2, B7	DRB3*0301(H)	0†	0	0	mDC	0	17	0
♀2	A2(H), B7(H)	DR15(H)	0	0	0	mDC	17	83	0
♀3	A2(H), B7	DQ5, DRB3*0301	0	0	0	-	50	33	0
♀4	A1, A2	DQ5	0	0	0	mDC	50	0	0
♀5	A2, B60(H)	-	0	0	0	-	63	0	0
♀6	A2, B7	DQ5	0	0	0	B, M, mDC, T, gran	75	50	25
♀7	A2(H)	DRB3*0301(H)	0	0	1	-	50	83	0
♀8	B7	DR15(H)	0	0	1	-	33	90	42
♀9	A1(H), B7	DR15(H)	0	0	1	-	0	75	0
♀10	A2	DR15(H)	0	0	3	-	67	50	33
♀11	A2, B7	DRB3*0301(H)	0	0	2	-	50	0	0
♀12	B7(H)	DR15(H)	0	0	2	-	42	50	0
♀13	A1, B8	-	0	0	4	-	40	*nt*	20
♀14	A2, B60	DRB3*0301(H)	1	0	0	-	67	0	0
♀15	-	DRB3*0301(H)	1∧	0	0	mDC	33	58	0
♀16	A1, A2, B60(H)	-	2	0	2	gran	75	20	20
♀17	A1	DR15(H)	2	0	4	T	100	25	50•
♀18	A2, B60(H)	-	2	0	4	B, M, mDC	20	*nt*	80•
♀19	A1, A2	DRB3*0301(H)	2	0	1 (+1TB)	-	67	17	0
♀20	-	DQ5, DRB3*0301(H)	3	1	0	T	57	100	29
♀21	A2(H)	DRB3*0301(H)	2	1	0	-	80	0	0
♀22	A2	-	3#	2	0	-	0	*nt*	0
♀23	A2, B7, B40	DRB3*0301(H), DQ5	4	3	0	T	67	67	0
♀24	B7(H)	DR15(H)	3	1	0	-	0	8	0
♀25	A2, B7(H)	DR15(H)	4	2	0	B	0	0	0
♀26	-	DR15(H)	2	2	0	-	50	0	0
♀27	B7	DR15(H)	1†	1	0	-	0	12	0
♀28	A2, B7	DR15	2	1	2	B	0	0	84•
♀29	A2	DQ5	1†	1	1	-	0	*nt*	*nt*
♀30	A2	-	2	2	3	-	67	*nt*	*nt*
♀31	A2	-	2	2	3	B, M, mDC	60	*nt*	*nt*
♀32	A2	-	1†	1	3	T	0‡	*nt*	14‡
♀33	B7	DRB3*0301(H)	3Δ	3	4	-	0	0	17
♀34	B7(H)	DR15(H)	2	1	2	-	67	17	33
♀35	B7(H)	DR15(H)	3	2	4	M, mDC	12	50	37
♀36	A1, B7	DR15(H)	3	2	3	B	50	0	33
♀37	B60(H)	-	1	1	1	B, mDC	25	*nt*	17
♀38	A2, B8	-	2	2	1	B	58‡	*nt*	0‡
♀39	A2	DQ5	2	2	1	-	10‡	*nt*	0‡
♀40	A1, A2, B8	DQ5	3	3	1	gran	50	33	33
♀41	A2,B60	DR15,DRB3*0301	1	1	5	B	0	40	0
♀42	A1, B7 (H)	DRB3*0301 (H)	2	2	2	T	20	0	0
♀43	B7	DR15 (H)	3	2	2	T	40	10	0
♀44	B7 (H)	DR15 (H)	2	1	3	B	0	20	0
♀45	A2 (H), B60	DRB3*0301	2	1	1	B, mDC	33	67	0

G* = Pregnancies enduring more than 16 weeks of gestational age;

@ inhibition of tetanus toxoid recall antigen induced footpad swelling in the presence of the relevant HY peptide measured in the tvDTH assay; donors displaying ≥50% reduction are defined as DTH regulator phenotype [7].

(H) = homozygous; TB = twin brother; mDC = myeloid dendritic cells, gran = granulocytes, T = T cells, B = B cells, M = monocytes.

† ≥1 early miscarriage (6–12 weeks of gestational age);

# miscarriage at 24 weeks (male fetus);

∧ elective abortion at 5 weeks; Δ elective abortion at 12 weeks.

*nt* = not tested.

‡ tested against a single HLA-A2/HY peptide instead of the HY^class-I^ peptide pool and another self minor Histocompatibility peptide instead of the HX peptide pool.

• excluded from Figure 3 on the basis of an unexplained profoundly suppressed swelling response in the presence of the control HX^class-I+II^ peptide pool.

HY^class-I^: HLA-A1/HY, HLA-A2/HY, HLA-B7/HY, HLA-B8/HY, HLA-B60/HY; HY^class-II^: HLA-DRB3*0301/HY, HLA-DR15/HY, HLA-DQ5/HY; HX^class-I+II^: HLA-A1/HX, HLA-A2/HX, HLA-B7/HX, HLA-B8/HX, HLA-B60/HX, HLA-DRB3*0301/HX, HLA-DR15/HX, HLA-DQ5/HX.

### Testing Treg function in the *trans vivo* Delayed Type Hypersensitivity Assay

**Figure 1:**
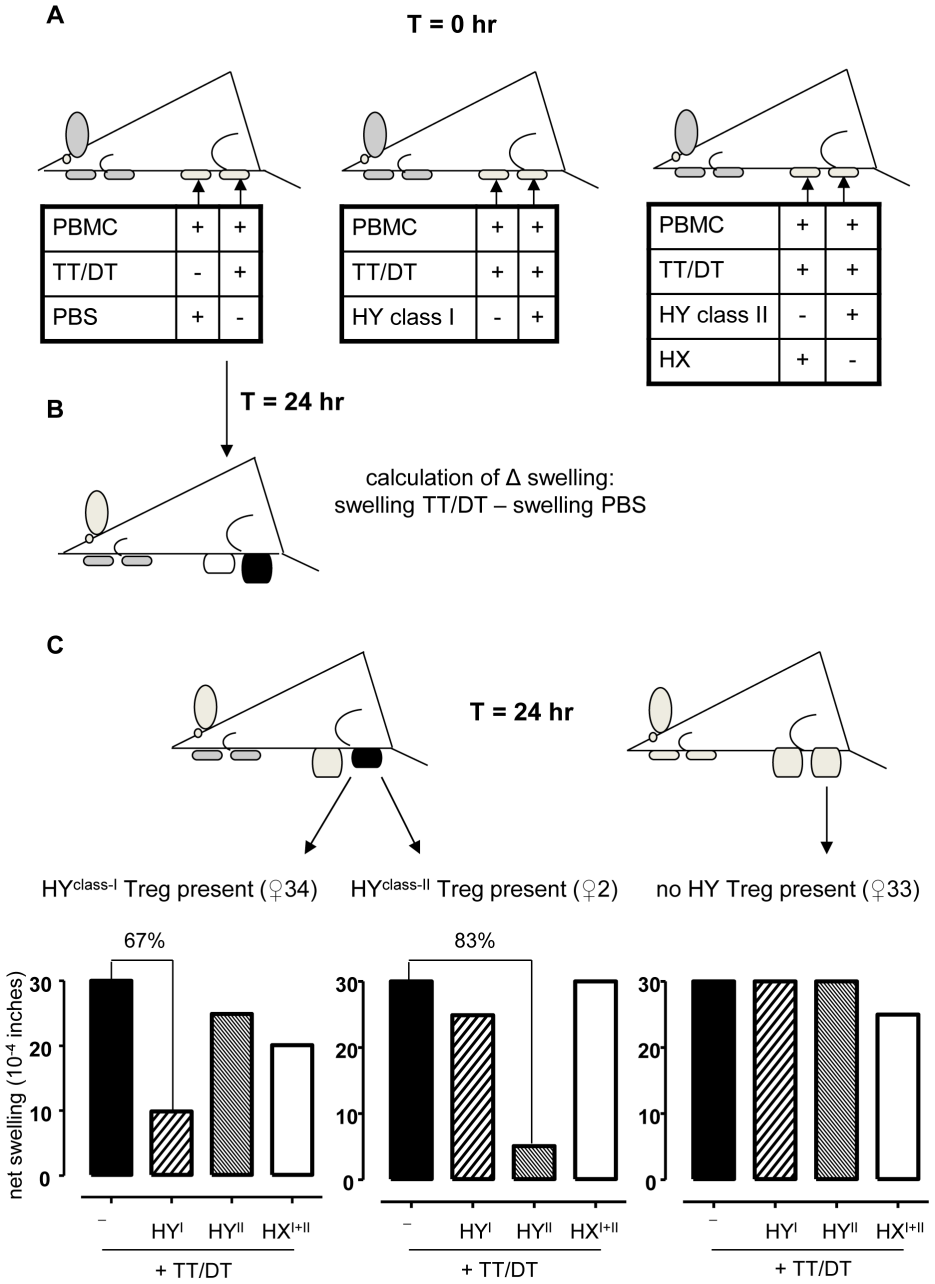
HLA class I or class II HY-specific Treg activity analyzed in the tvDTH assay. A) Outline of the complete set of footpad injections performed at T = 0 hour which are necessary for demonstrating the presence, or absence, of HY-specific Treg. B) Injection of PBMC and TT/DT recall antigens into the right footpad at T = 0 hour leads to a classical delayed type hypersensitivity response when donors have been pre-exposed to these antigens for instance through vaccination. This leads to swelling of the right footpad depicted in black which is measured at T = 24 hour. The left footpad serves as a control for calculation of the net swelling response. C) When Treg are present in the PBMC and properly activated, these cells inhibit the recall antigen-induced footpad swelling leading to only marginally swollen footpads as the black left footpad of the left mouse. When Treg are not present, or present but not appropriately activated when control HX peptides are co-injected, the footpad swelling response is comparable to the positive control swelling response induced by the recall antigens (gray right footpad of the mouse depicted on the right). From the net swelling responses, the percentage of inhibition of the recall antigen-induced response (depicted by the black bars) in the presence of respectively pooled HLA class I restricted HY peptides (HY^I^, hatched bars), pooled HLA class II restricted HY peptides (HY^II^, finely hatched bars) or control HX peptides (HX^I+II^, white bars) can be calculated as shown by the bar graphs. These graphs show representative data from two women which display HY-specific Treg activity (♀2 and 34). The percentage inhibition of the recall antigen-induced net swelling is plotted in each graph. The right graph depicts the results of donor ♀33 who displays no regulation against either the HY^class-I^, the HY^class-II^ or the control HX^class-I+II^ peptide pools.

T cell receptor-induced activation of Treg can result in the suppression of T cells with the same or other specificities [11]. The latter suppressive mechanism is generally referred to as linked suppression and can be specifically addressed in the *trans vivo* Delayed Type Hypersensitivity (tvDTH) assay [12]–[14]. The reproducibility of this assay has been described elsewhere [15]. As depicted in Figure 1, this experimental set up independently triggers Treg and responder T cells co-present in PBMC with respectively HY peptides and well defined recall antigens. The tvDTH assay was earlier applied to determine the presence of antigen-specific Treg after triggering their T cell receptor with a single synthetic peptide [7], [13], [16]. In the present study, three peptide pools were prepared comprising equal amounts of respectively 5 HLA class I-restricted HY peptides (HY^class-I^), 3 HLA class II-restricted HY peptides (HY^class-II^), and 8 corresponding X homologue peptides (HX^class-I+II^). The amino acid sequences of these peptides are listed in Table 2 [17]. As indicated in Table 1, some donors were tested against a single HY peptide (HLA-A2/HY) [7] and a self minor H antigen instead of the HY or HX peptide pool. As visualized in Figure 1B and 1C, footpad swelling, induced after chemokine release by activated recall antigen-specific CD4^pos^ memory T cells, was measured 24 hours after the co-injection of PBMC and recombinant Tetanus Toxoid (TT) and Diphtheria Toxoid proteins into the hind footpads of naïve CB17.SCID mice [18]. The net swelling response of each footpad is subsequently calculated by distracting footpad swelling induced by injecting PBMC+PBS from the response induced in the presence of antigens (Figure 1B). The presence of HY peptide-specific Treg is defined when the net swelling response is inhibited by ≥50% in the co-presence of HY peptides but not in the co-presence of control HX homologue peptides or a control self minor H antigen (Figure 1C, left and center plot) [7], [13], [15]. In addition, each donor was also tested for footpad swelling induced by the combination of PBMC+alloantigen alone, respectively the HY^class-I^, HY^class-II^ and HX^class-I+II^ peptide pool. Note that the tvDTH assay was performed in a blinded fashion, i.e. the details on the womens' chimeric status, pregnancy history and supplied peptide pools were revealed after all samples had been analyzed.

**Table 2:** HLA class I and class II HY and homologue HX peptide sequences.

HLA-restriction site	HY-peptide	HX-peptide
HLA-A1	IVD**C** *LTEMY	IVD**S**LTEMY
HLA-A2	FI**D**SY**I**C**QV**	FI**E**SY**V**C**RM**
HLA-B7	SP**S**VDKA**R**AEL	SP**A**VDKA**Q**AEL
HLA-B8	LPHN**H**T**D**L	LPHN**R**T**N**L
HLA-B60	**R**ESEE**E**S**V**SL	**G**ESEE**A**S**P**SL
HLA-DRB3*0301	**RTIRYPDPV**IKVNDT**V**QID**LGT**	**L**IKVNDT**I**QID
HLA-DR15/HY	ASKGRYIPPHLRNKEA**SK**GF	ASKGRYIPPHLRN**R**EA**TR**GF
HLA-DQ5	TG**S**NCPPHIE**N**FSD**ID**MGEI	TG**N**NCPPHIE**S**FSD**VE**MGEI

Amino acid difference between HY and HX peptide are indicated in bold;

* = cystinylated amino acid.

### Isolation of leukocyte cell types

**Figure 2:**
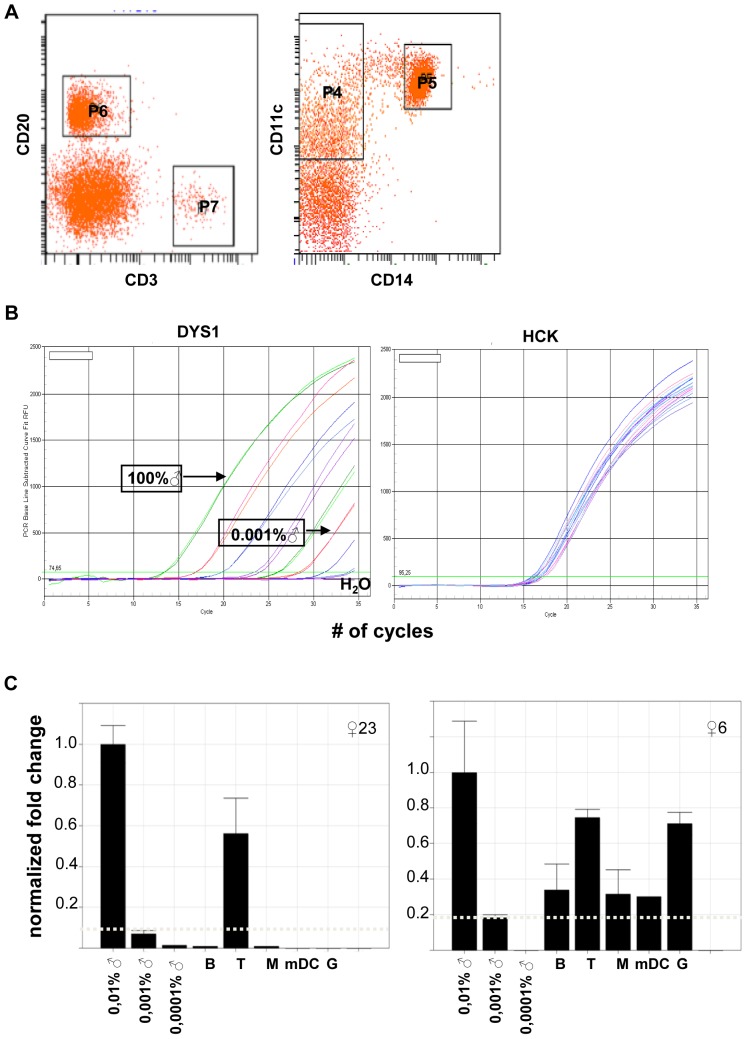
Isolation of leukocyte subsets by FACS cell sorting and detection of microchimerism by qPCR. A) A representative example of the simultaneous isolation of CD3^pos^ T cells (P7), CD20^pos^ B cells (P6), CD14^pos^ monocytes (P5) and CD3^neg^CD20^neg^CD14^neg^CD11c^pos^ myeloid Dendritic Cells (P4) from PBMC which had been depleted for T cells by incubation with CD3 MACS beads prior to 4 color FACS sorting. B) Addressing the sensitivity of MC^Y-gene^ detection by qPCR using a standard curve comprising DNA isolated from a 10-fold dilution series prepared with a male Epstein Barr Virus-transformed lymphoblastoid cell line (EBV-LCL) diluted in a female EBV-LCL (left plot). Each test sample was simultaneously amplified with HCK-specific primers (right plot) for data normalization as explained in the materials and methods section. C) To representative sets of chimerism data. The left plot shows the data obtained from a multiparous woman (♀23) who displays MC^Y-gene^ only in isolated T cells (T), but not in B cells (B), monocytes (M), myeloid Dendritic cells (mDC) or granulocytes (gran); the right plot displays the data from a nulliparous woman (♀6) in whom MC^Y-gene^ was detected in multiple cell types. The grey horizontal dotted line represents the limit of detection of the applied qPCR assay. The normalized fold change depicted at the Y-axis indicates the level of microchimerism adjusted to the assay's control DNA sample (1 male EBV-LCL in 10.000 female EBV-LCL = 0,01% ♂), which is arbitrarily put at 1.

Peripheral blood mononuclear cells (PBMC) were isolated using Ficoll-Isopaque density gradient centrifugation. Granulocytes were obtained from the remaining cell pellet after lysing red blood cells with NH_4_Cl and KHCO_3_ containing lysis buffer. CD3^pos^ T cells were obtained from PBMC using the CD3 MACS isolation procedure according to manufacturer's instructions (Miltenyi Biotec, Bergisch Gladbach, Germany). The remaining cells were subsequently labeled with FITC, PE, PerCP or APC conjugated antibodies (all from BD Biosciences, Amsterdam, the Netherlands) for isolation of CD20^pos^ B cells, CD14^pos^ monocytes and CD3^neg^CD20^neg^CD14^neg^CD11c^pos^ myeloid Dendritic Cells (mDC) by flow cytometric cell sorting on a FACS-ARIA cytometer (Becton Dickinson) (Figure 2A).

### Detection of circulating male microchimeric cells

Isolation of genomic DNA from total PBMC and from purified leukocyte subsets was performed as previously described [7] using the QIAmp DNA blood minikit (QIAGEN). Note that DNase-free RNase (Roche) was present during protein digestion at 56°C. The concentration of each DNA sample was quantified on a NanoDrop 1000 spectrophotometer (Thermo Scientific). The presence of male DNA (indicating the presence of MC^Y-gene^) was assessed by amplification of a multi-copy Y chromosome-specific DNA sequence (*DYS1*) using quantitative real-time PCR (qPCR). Amplification reactions were performed with 750 ng of DNA and 25 µl IQ Supermix (200 nM forward primer, 200 nM reverse primer, 200 nM probe, Biorad) in a final reaction volume of 50 µl. Template DNA was amplified and quantified in a MyiQ single color real-time PCR detection system (BioRad) under the following conditions: 95°C for 10 minutes, 5 cycles at 95°C for 60 seconds, 60°C for 30 seconds and 72°C for 30 seconds, followed by 35 cycles at 95°C for 15 seconds, 60°C for 30 seconds and 72°C for 30 seconds. The number of quantification cycles (Cq) [19] were calculated using iQ5 optical software (Bio-Rad). To standardize for putative variation in DNA input and in amplification efficiency, a second PCR detecting the human hematopoietic cell kinase (*HCK*) reference gene was performed in parallel [20]. Primer and probe sequences (Sigma-Genosys Ltd) are listed in Table 3. Every PCR experiment included template controls (H_2_O) for each reaction and a standard curve, prepared from DNA isolated from dilution series of male Epstein Barr Virus transformed lymphoblastoid cell lines (EBV-LCL) diluted in female EBV-LCL (Figure 2B), was used to determine the limit of detection (LOD). Male DNA is reliably detected at the level of 1 male cell diluted in 10^5^ female cells ( = 0,001%) as depicted by the horizontal line in Figure 2C. Each sample was tested in duplo and at least in two independently performed assays to correct for possible interrun variation. Only samples in which *DYS1* amplification consistently occurred at or before 35 Cq qualified for data analysis. Processed data are depicted as normalized fold change compared to the same reference DNA sample (one male cell in 10^4^ female cells which is arbitrarily set to 1). For validation purposes, some randomly selected samples were tested in parallel by a different qPCR assay specific for *DYS1* as described [21]. Female donors were scored as MC^Y-gene^ positive when male DNA could be detected at or above the LOD in at least one purified leukocyte cell type in two independent qPCR experiments.

**Table 3:** Primer and probe nucleotide sequences used for the detection of Y chromosome positive microchimeric cells by real-time PCR.

primer	nucleotide sequence	fragment size
Forw-DYS1	5′-TCCTGCTTATCCAAATTCACCAT- 3′	86 bp
Rev-DYS1	5′- ACTTCCCTCTGACATTACCTGATAATTG-3′	
Y probe	5′- (FAM)-AAGTCGCCACTGGATATCAGTTCCCTTGT- (TAMRA)-3′	
Forw-HCK	5′-TATTAGCACCATCCATAGGAGGCTT-3′	81 bp
Rev-HCK	5′-GTTAGGGAAAGTGGAGCGGAAG-3′	
HCK Probe	5′- (FAM)-TAACGCGTCCACCAAGGATGCGAA- (TAMRA)-3′	

### Statistical analyses

For statistical analysis of the data IBM SPSS Statistics 20 was used. Statistical significance was defined by p-value<0.05.

## Results

### Presence of Treg specific for HLA class I- or HLA class II-restricted HY peptides is not restricted to women with an obstetric history

**Figure 3:**
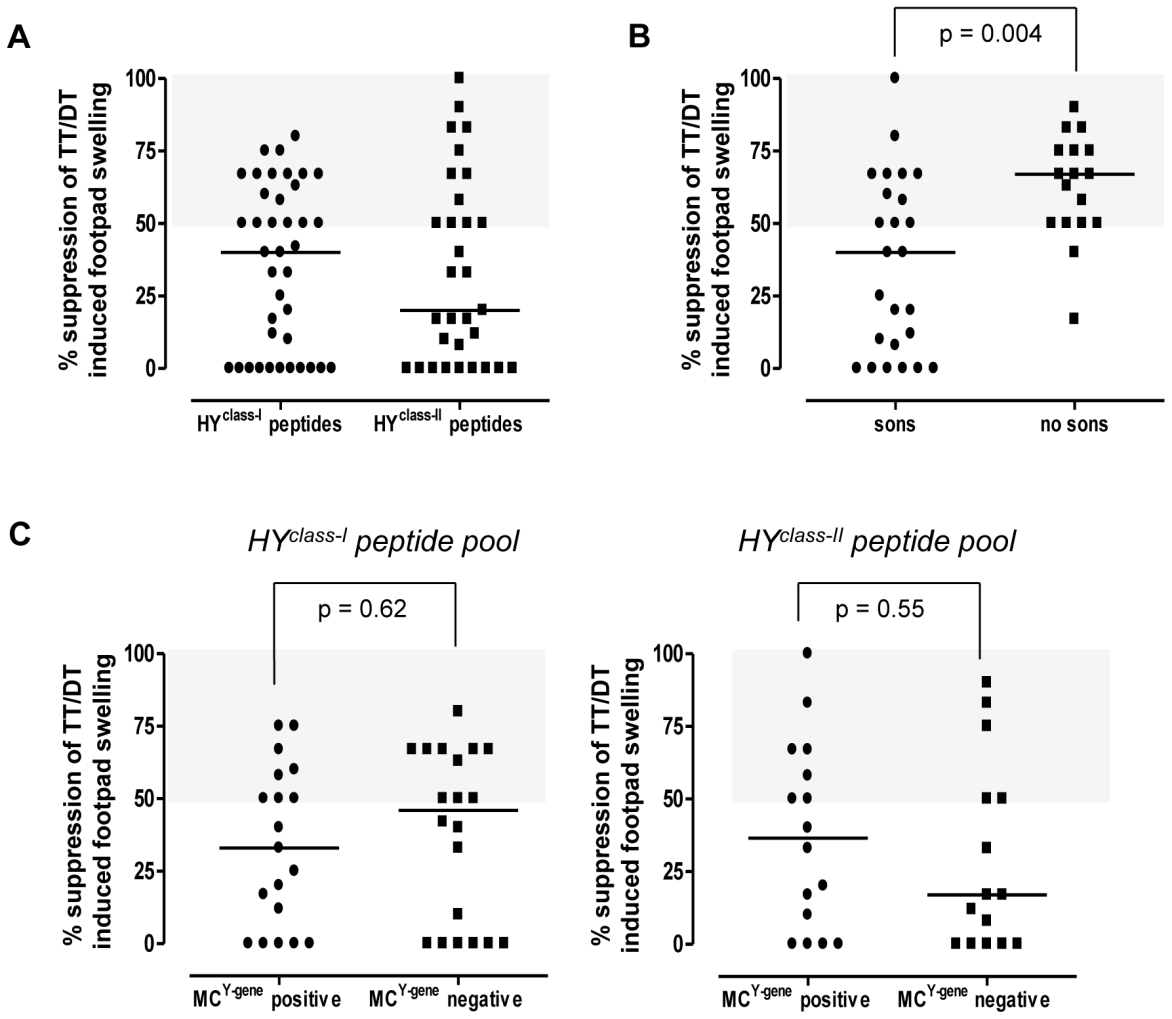
tvDTH regulation and microchimerism in healthy women. A) tvDTH regulation induced by either pooled HLA class I restricted HY peptides (HY^class-I^) in n = 39 donors (left column) or by pooled HLA class II restricted HY peptides (HY^class-II^) in n = 31 donors. Donors with demonstrable Treg function, defined as described in Material and Methods section, are displayed in the light gray areas of each graph. B) Comparison of HY peptide-specific regulation of women without sons (left column, n = 17) with women with sons (n = 25). Corresponding statistical data analysis of the two groups is shown in Table 4 (Pearson Chi-square, two-sided p = 0.004). Depicted are the highest levels of HY^class-I^ and/or by HY^class-II^ peptide induced suppression measured in each donor. C) Comparison of the presence/absence of circulating male microchimeric leukocytes (MC^Y-gene^ positive/MC^Y-gene^ negative respectively) with the presence/absence of HLA class I and/or class II HY specific Treg. The left panel shows the percentage suppression of the Tetanus Toxoid/Diphtheria Toxoid (TT/DT) recall antigen-induced footpad swelling in the presence of pooled HLA class I restricted HY peptides (HY^class-I^ peptide pool); the right panel shows suppression of the TT/DT recall antigen-induced footpad swelling in the presence of pooled HLA class II restricted HY peptides (HY^class-II^ peptide pool). Corresponding statistical data analysis (Pearson Chi-square, two-sided) is shown in Table 5.

PBMC from 45 women were tested in the tvDTH assay against the relevant HY peptide pools. Three women (♀17, 18, 28) showed high regulatory responses against the control HX peptide pool. Twenty-six of the remaining 42 women (62%) showed HY peptide-specific suppression. Specifically, 18 out of 39 (46%) women expressing at least one of 5 HY peptide-binding HLA class I molecule displayed HY^class-I^ peptide-induced suppressive function when the recall antigen and a pool containing 5 different HLA class I-restricted HY peptides were co injected (Table 1, Figure 3A). Twelve out of 31 women (39%), expressing at least one of the 3 HY peptide-binding HLA class II molecules, displayed HY^class-II^ peptide-induced suppressive function when the recall antigen and a pool containing 3 different HLA class II-restricted HY peptides were co injected (Table 1, Figure 3A). From the group of 28 women expressing both relevant HLA class I and class II molecules, four (14%) displayed HY-specific regulation against both the class I and class II HY peptide pool. None of the women tested in the tvDTH assay displayed substantial footpad swelling (earlier defined as a net swelling of ≥10×10^−4^ inches [13]) when their PBMC were injected with HY or HX peptides alone (data not shown).

Next, the tvDTH data were stratified according to the womens' pregnancy history and, if applicable, to the sex of their offspring. Strikingly, the presence of the HY-specific Treg was not only restricted to women who had given birth to male offspring (Figure 3B, Table 1 and 4). A significantly higher percentage of women without sons (15 out of 17, 88%) showed HLA class I and/or class II HY-peptide specific regulation compared to 11 out of 25 (44%) women who have sons (Table 4, Pearson Chi-square, two-sided p = 0.004).

**Table 4:** Correlation between women without male offspring and the presence of Treg.

	tvDTH Treg		p-value*
	*Yes*	*No*	
**Male offspring**			
*Yes*	11	14	0.004
*No*	15	2	

* Pearson Chi-square test, two-sided.

### Male microchimerism in myeloid and lymphoid lineage-derived cells

The presence of MC^Y-gene^ was determined in fractionated leukocytes obtained from all 45 women included in this study (Table 1). MC^Y-gene^ could be detected in 24 out of 45 (53%) women. Male DNA was detected in single types of lymphoid cells, i.e. in T cells (n = 6) in B cells (n = 6), in myeloid DC (n = 4), in granulocytes (n = 2) and also simultaneously in more than one of these cell types (n = 6) [22]. The qPCR results obtained from two representative sets of samples are depicted in Figure 2C. Male DNA was detectable in circulating leucocytes collected between 2 (♀28) and 35 (♀38) years after delivery of male offspring in 16 out of 26 women (62%). MC^Y-gene^ could also be detected in 8 out of 19 (42%) women without male offspring. Of the latter 8 women, two may have carried a male fetus as they experienced a spontaneous miscarriage (♀1) and an elective abortion (♀15) during the first trimester of pregnancy (Table 1). Three out of the 8 women without male offspring reported the delivery of only female offspring after uncomplicated pregnancies (♀16, 17 and 18, Table 1). Note however that the latter 3 women had an older brother.

### The presence of HY-specific Treg is independent of the detection of circulating male cells

We next stratified the tvDTH data according to the detectable presence (n = 24) or absence (n = 21) of circulating male cells (Figure 3C). No statistically significant correlation was found between the presence of either HLA class I (p = 0.62)- or class II (p = 0.55)-restricted HY-specific Treg and the presence of circulating MC^Y-gene^ bearing cells. Neither did we see an association between the presence of male DNA and male offspring in the respective women (Table 5).

**Table 5:** No correlation between MC^Y-gene^ positive women and women with or without male offspring neither with women with Treg.

	MC^Y-gene^		p-value*
	*Positive*	*Negative*	
**Male offspring**			
*Yes*	16	10	0.18
*No*	8	11	
**tvDTH Treg**			
*Yes*	13	13	1.00
*No*	8	8	
**tvDTH Treg class I**			
*Yes*	8	10	0.62
*No*	11	10	
**tvDTH Treg class II**			
*Yes*	7	5	0.55
*No*	9	10	

* Pearson Chi-square test, two-sided.

## Discussion

Using a well-defined cohort of healthy female blood donors, we assessed whether the long term presence of HY-specific Treg is restricted to women with male offspring and whether its presence is associated with the presence of HY antigen-expressing microchimeric cells. With regard to the former, two observations were made. First, HY-specific Treg were observed in 11 out of 25 women who delivered a son. This finding is in line with our earlier findings in 4 women [7]. Although not analyzed in the underlying study, we assume that Treg negative women with male offspring bear memory T cells specific for the HY antigens. Our previous data and those of other investigators, in which HY-specific CTL have been isolated from peripheral blood of women with sons, favor this assumption [4]–[7]. Anticipating on the latter assumption, the precursor frequencies of these cells must be quite low given that injection of PBMC and HY peptides did not result in substantial footpad swelling responses when compared to responses induced by classical recall antigens such as TT and DT. Given the substantial variation in tvDTH responses (Figure 3A), varying from profound to only marginal or no suppression of footpad swelling responses, it is likely that HY-specific CTL co-exist in a proportion of the women with male offspring analyzed in this study.

In addition to the demonstration of HY-specific Treg activity in a substantial proportion of this female cohort, three women (♀17,18 and 28) displayed profoundly suppressed recall antigen-induced footpad swelling response (≥50% reduction) when the recall antigens were co-administered with the control HX peptide pool. Seven women displayed limited reduction (25–50%) while 32 women showed no reduction (0–25%) of footpad swelling in the co-presence of HX-peptides. We have as yet no explanation for these observations.

The second and surprising observation was the detection of HY-specific Treg in 15 out of 17 women without male offspring (Table 1 and 4). This observation is in line with our recent report on the presence of HY-specific CTL in cord blood cells from newborn girls without an older brother [9]. The origin of male cells was not traced in these babies. The observations in the current study further support the concept of transmaternal flow of, amongst others, male cells from an as yet unknown source [9], [23].

The MC^Y-gene^ analyses in the current study showed that the presence of male microchimerism was frequent in women with male offspring (16 of 26, 62%, Table 5). Similar to the study of Yan *et al* [24], MC^Y-gene^ was also detected in 4 out of 13 (31%) nulliparous women tested in our study (Table 1). Three of the 8 MC^Y-gene^ positive women without male offspring indeed had an older brother (♀16, 17 and 18), while only the mother and/or grandmother of 3 other women (♀2, 4 and 6) of this group had older brother(s). Although this issue needs to be addressed in a larger study population, one may conclude that transmaternal passage of male cells, and thus establishment of male microchimerism, might occur even across generations. Possible other sources of MC^Y-gene^ could be male leukocytes present in semen entering the female's circulation [25], a ‘vanished’ male twin brother [10] and (un)known miscarriages of male fetuses [26].

We analyzed whether or not the presence of male chimerism is a crucial condition for the persistence of HY-specific Treg. Although the dataset is relatively small to draw definitive conclusions, neither the presence of HY^class-I^ peptide-specific Treg nor of HY^class-II^ peptide-specific Treg could be associated with the co-presence of circulating male microchimeric cells (Figure 3C, Table 5). Likewise, HY-specific Treg activity could not be related to any particular microchimeric cell type. Note that the latter conclusions are based on the detection limit of the applied qPCR (Figure 2B,C). Additionally, the detection of male microchimerism may be influenced by a day-to-day variation in cellular output by the bone marrow. It is known that small numbers of male hematopoietic stem cells hiding in female bone marrow niches serve as a persisting source of male antigens [27]. Furthermore, we did not address the level of tissue-residing MC^Y-gene^ expressing cells [28], [29]; the latter type of microchimeric cells was found to be critically important for the induction of murine alloreactive Treg [30].

Of note, six women (♀6, 18, 31, 35, 37 and 45) displayed MC^Y-gene^ in more than one leukocyte cell type derived from either the myeloid or lymphoid lineage. In addition to the earlier reported presence of male progenitor cells in parous women with male offspring [27], we observed a multi-lineage chimerism pattern in three consecutive blood samples collected from ♀31 over a period of 8 years (data not shown) suggesting the presence of male hematopoietic progenitor cells. Four out of these 6 women had male offspring, and show HY-specific Treg as demonstrated in the current and in an earlier study from our group [7].

In conclusion, we here report the overall presence of HLA class I- and II-restricted HY-specific Treg in healthy female blood donors. Additional functional characterization of the latter Treg is subject of future studies. Limited earlier results in healthy subjects demonstrated the expression of Treg-associated markers such as CTLA-4 [7], [31] by minor H antigen-specific Treg. Information on the role of HY-specific Treg in HSCT is virtually non-existent. The one clinical study that deals with minor H antigen-specific Treg is reported in the setting of renal transplantation [31]. Here, CD8^pos^ CTL and CD8^pos^ Treg specific for the same autosomal minor H antigen HA-1 coexisted in a tolerant long term renal allograft. In the tvDTH assay, HA-1-specific Treg showed TGF-β mediated suppression of HA-1 peptide-specific responses [31]. HY-specific Treg appear to be rather common in the women enrolled in our study. Their prevalence is not confined to women who have carried a male fetus, since HY-specific Treg can be demonstrated in women without male offspring as well. The presence of HY-specific Treg is not necessarily accompanied by the presence of male microchimerism. The possible clinical relevance of our study for HSCT may be found in female donors with HY-specific CTL. Since HY antigens are broadly expressed, the simultaneous presence of HY-specific Treg may well diminish severe Graft-versus-Host immune reactivity in male HSCT recipients.
